# Exploring the effects of doxorubicin on survival rates in glioma patients: a comprehensive systematic review

**DOI:** 10.1186/s40001-025-02674-5

**Published:** 2025-05-28

**Authors:** Elyar Mahboubi, Mahdieh Mondanizadeh, Amir Almasi-Hashiani, Elham Ghasemi

**Affiliations:** 1 Department of Biotechnology and Molecular Medicine, School of Medicine, Arak University of Medical Sciences, Arak, Iran; 2 Department of Epidemiology, School of Health, Arak University of Medical Sciences, Arak, Markazi Islamic Republic of Iran; 3 Traditional and Complementary Medicine Research Center, School of Medicine, Arak University of Medical Sciences, Arak, Iran

**Keywords:** Glioma, Doxorubicin, Overall survival, Progression-free survival

## Abstract

**Background:**

Glioma is still one of the most aggressive and common types of brain and central nervous system cancer, with few effective treatment options. Despite progress in therapy, there is a requirement for new methods to enhance patient outcomes. Doxorubicin (DOX), a type of anthracycline that has been proven to be effective in different cancers, encounters difficulties in treating glioma because of the blood–brain barrier. This systematic review is intended to assess the effects of DOX on the survival and quality of life of glioma patients.

**Methods:**

We conducted a thorough search and found 1576 records, from which 10 studies met our inclusion criteria. These studies, published between 1973 and 2023, were examined for overall survival (OS), progression-free survival (PFS), median time to progression (mTTP), response rate, and toxicity profiles.

**Results:**

The studies included in the analysis showed that OS ranged from 6 to 18.5 months, indicating potential improved outcomes with liposomal and PEGylated forms of DOX. PFS rates varied from 15 to 58%, and mTTP ranged from 11 to 39.83 weeks. Response rates varied from 19 to 88%, and while DOX was generally well-tolerated, some hematological and non-hematological toxicities were observed. DOX shows promise in prolonging survival and slowing glioma progression, especially with advanced delivery methods.

**Conclusions:**

However, variations in research, small sample sizes, and lack of uniformity highlight the need for further investigation. Overcoming these limitations in future studies is crucial to maximize DOX's effectiveness in glioma treatment and improve patient outcomes.

## Introduction

Glioma remains the most aggressive and commonly occurring form of brain and central nervous system tumor despite advancements in cancer treatment [1]. Gliomas are a group of tumors that develop in the central nervous system from glial cells. They are classified into four groups—I to IV—based on their level of malignancy according to the guidelines of the World Health Organization (WHO) [2]. The most prevalent form of brain cancer in the nervous system constitutes more than 80% of all primary malignant brain tumors, affecting approximately 10,000 individuals in the USA and 74,000 people globally each year [3]. 

The typical treatment approach for glioma involves performing the most extensive and safe surgical removal possible, followed by a combination of radiotherapy and chemotherapy using temozolomide [4]. Researchers are searching for innovative methods to overcome glioma's resistance to initial treatment, experimenting with various approaches, including the use of tumor treating fields and adjuvant therapy with drugs, such as BCNU, CCNU, bevacizumab, and others, to improve OS and quality of life for glioma patients [4, 5].

DOX is a drug that has garnered the attention of scientists. It is an efficient derivative of anthracycline, also known as Adriamycin, extracted from Streptomyces peucetius [6]. Since the 1960 s, this medication has been commonly prescribed as a chemotherapy treatment for breast cancer, ovarian cancer, sarcoma, lymphoma, and various other forms of cancer [6, 7]. This anti-neoplastic drug acts through the DNA intercalation, topoisomerase II inhibition and generating reactive oxygen species (ROS) to stop the growth of cancer cells and trigger p53-mediated cell death [8]. DOX shows promising potential for use as a standalone treatment or in combination with other types of glioma therapies. However, significant challenges exist in utilizing this drug against glioma, with the biggest obstacle being the blood–brain barrier. Researchers are employing various methods, such as pegylated liposomal DOX and nanocarriers, to address these barriers [9, 10].

Numerous clinical trials and observational studies have been conducted to explore the impact of DOX on disease-free survival (DFS), PFS, median overall survival (MOS), and the quality of life of glioma patients. However, a comprehensive study systematically examining all these outcomes has been lacking. Therefore, we conducted a systematic review to analyze the different aspects of using DOX in improving survival rates and the quality of life for glioma patients. 

## Materials and methods

### Study design

This systematic review was conducted following the Preferred Reporting Items for Systematic Reviews and Meta-Analyses (PRISMA) guidelines [11]. We aimed to evaluate the impact of DOX on the survival and quality of life of glioma patients. The review process included a comprehensive literature search, study selection, data extraction, and quality assessment. All methods were carried out in accordance with relevant guidelines and regulations. This study was approved by the local Medical Ethics Committee of Arak University of Medical Sciences with identification code: IR.ARAKMU.REC.1402.232. 

### Search strategy

We performed an extensive search of electronic databases, including PubMed, Web of Science, and Scopus, from their inception until September 2023. In addition, we utilized Google Scholar to search for gray literature. The search strategy was developed using a combination of medical subject headings (MeSH) and other terms related to glioma and DOX. The search terms used in this study are presented in Table 1.

**Table 1:** Search query in databases that included in the study

Databases	Query
Web of science	"Disease-Free Survival"(All Fields) or"Survival Rate"(All Fields) or"Progression-Free Survival"(All Fields) or"Survival"(All Fields) or"Mortality"(All Fields) AND"DOX"(All Fields) or"Rubex"(All Fields) or"Adriamycin"(All Fields) or"Adriblast*"(All Fields) or"Farmiblastina"(All Fields) or"Ribodoxo"(All Fields) AND"Glioma"(Topic) or"Gliomas"(All Fields) or"Glial Cell Tumor"(All Fields) or"Mixed Glioma"(All Fields) or"Malignant Glioma"(All Fields)
PubMed	("Disease-Free Survival"[MeSH Terms] OR"Survival Rate"[MeSH Terms] OR"Progression-Free Survival"[MeSH Terms] OR"Survival"[MeSH Terms] OR"Disease-Free Survival"[All Fields] OR"Survival Rate"[All Fields] OR"Progression-Free Survival"[All Fields] OR"Survival"[All Fields] OR ("Mortality"[MeSH Terms] OR"Mortality"[All Fields])) AND ("Glioma"[MeSH Terms] OR"Glioma"[Text Word] OR"Gliomas"[Text Word] OR"Glial Cell Tumor"[Text Word] OR"Mixed Glioma"[Text Word] OR"Malignant Glioma"[Text Word]) AND ("DOX"[MeSH Terms] OR"DOX"[All Fields] OR"DOX"[Text Word] OR"Rubex"[All Fields] OR"Adriamycin"[All Fields] OR"adriblast*"[All Fields])
Scopus	((TITLE-ABS-KEY ("Disease-Free Survival") OR TITLE-ABS-KEY ("Survival Rate") OR TITLE-ABS-KEY ("Progression-Free Survival") OR TITLE-ABS-KEY ("Survival") OR TITLE-ABS-KEY ("Mortality"))) AND ((TITLE-ABS-KEY ("DOX") OR TITLE-ABS-KEY ("Rubex") OR TITLE-ABS-KEY ("Adriamycin") OR TITLE-ABS-KEY ("Adriblast*") OR TITLE-ABS-KEY ("Farmiblastina") OR TITLE-ABS-KEY ("Ribodoxo"))) AND ((TITLE-ABS-KEY ("Glioma") OR TITLE-ABS-KEY ("Gliomas") OR TITLE-ABS-KEY ("Glial Cell Tumor") OR TITLE-ABS-KEY ("Mixed Glioma") OR TITLE-ABS-KEY ("Malignant Glioma")))

### Selection criteria

The search and screening for this study were independently conducted and evaluated by E.M and E.G. The inclusion and exclusion criteria were established based on the PICO framework: participants were individuals with various subtypes of human glioma, Intervention was administration of the chemotherapeutic drug DOX, Comparison was between patients who received DOX and those who did not and the Outcome was assessment of the impact of DOX on the survival and quality of life of glioma patients.

The inclusion criteria of this study were: (1) studies examining the survival rate of glioma patients treated with DOX, (2) original research articles, (3) articles written in English, (4) no restriction on publication date, and (5) studies involving human participants.

The exclusion criteria were: (1) letters, case reports, reviews, and conference abstracts, (2) non-English papers, (3) methodological studies, and (4) studies not specifically focused on glioma patients.

### Data extraction process

Two reviewers (E.M. and E.G.) independently screened titles and abstracts of all identified studies for eligibility. Full texts of potentially relevant articles were then reviewed to confirm their eligibility based on the inclusion and exclusion criteria. Discrepancies were resolved through discussion or consultation with a third reviewer. Data extraction was performed independently by E.M. and subsequently verified by E.GH. The following data were extracted from each included study: author(s) name(s), year of publication, study location, sample type and size, study design, WHO classification of glioma, gender distribution, mean age of participants, disease characteristics, DOX dosage and administration details, survival outcomes (e.g., OS, progression-free survival, and median time-to-progression), response rates, and toxicity.

### Quality assessment

The quality of included studies was assessed independently by two reviewers (E.M. and E.G.) using the checklists, such as Newcastle–Ottawa Scale (NOS) for cohort studies, Cochrane Risk of Bias for randomized controlled trials (RCTs) and JBI Checklist for case series. Disagreements were resolved by consensus or consultation with a third reviewer. Heterogeneity was assessed qualitatively, and due to variations in study designs and outcome measures, a meta-analysis was not performed. The assessment focused on selection, comparability, and outcome domains to ensure the reliability and validity of the findings.

### Data synthesis and analysis

A narrative synthesis of the findings was conducted, with a focus on the impact of DOX on OS, progression-free survival, median time-to-progression, response rates, and toxicity. Quantitative data were summarized using descriptive statistics. Due to the heterogeneity in study designs and outcomes, a meta-analysis was not performed.

## Results

### Study selection and characteristics

**Fig. 1:**
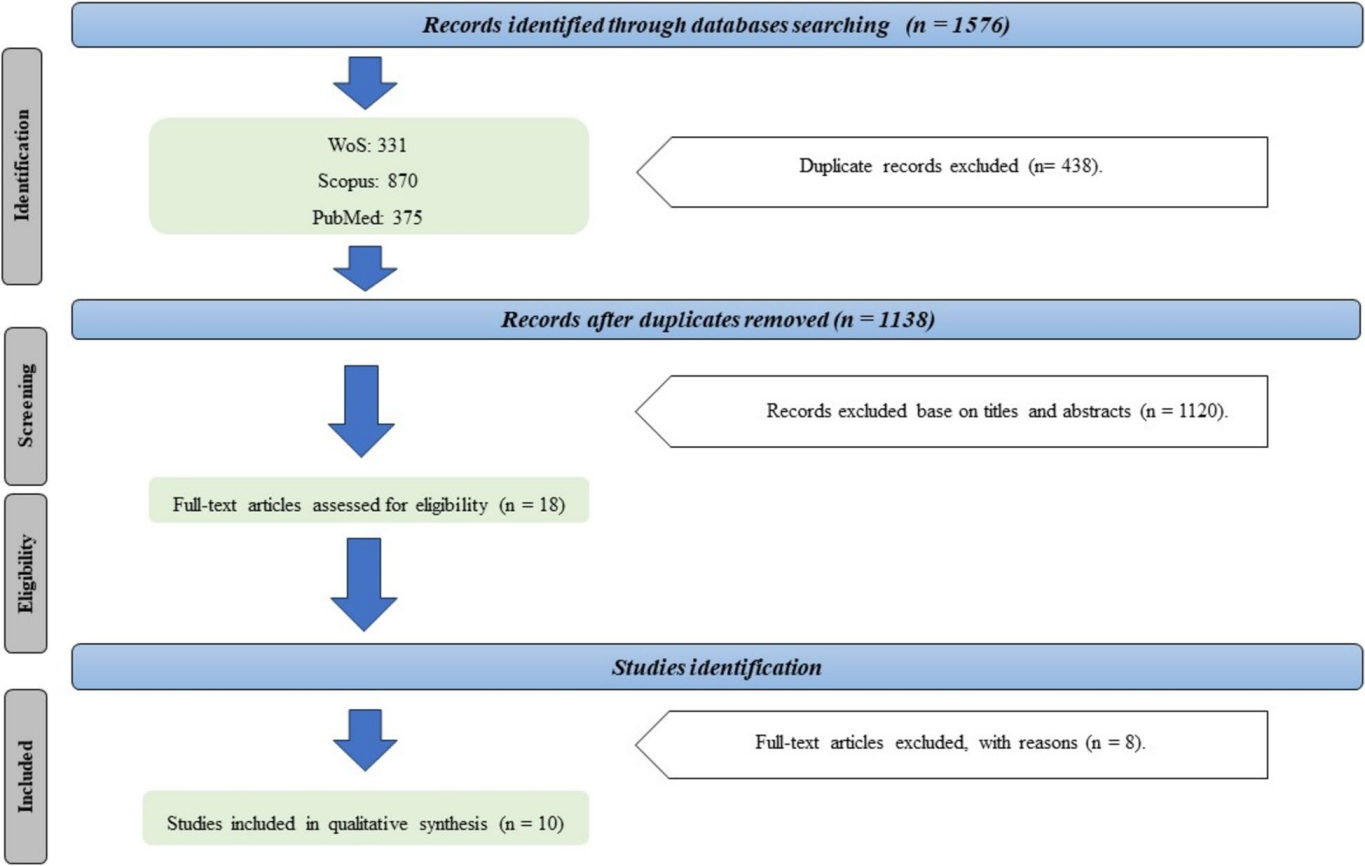
Screening flowchart for selecting articles in this systematic review

Initially, 1,576 records were identified at the start of the search. After eliminating 438 duplicates, 1,120 publications were excluded based on their titles and abstracts not being relevant. Next, 18 full-text articles were assessed, and 8 articles were excluded for reasons, such as not meeting the necessary criteria or lacking available related data. Ultimately, 10 articles were included in this systematic review based on the aforementioned inclusion and exclusion criteria. Figure 1 presents a summary of the findings obtained from the eligible articles.

The selected studies primarily examined the impact of DOX on the survival rate of patients. Among these studies, eight focused on patients with WHO grade IV glioma, while two included patients with WHO grade IV and III gliomas. This systematic review encompassed studies published from 1973 to 2021, with varying case sample sizes ranging from 7 to 63 (totaling 366 cases), most of which were conducted in Germany. Our analysis included 10 studies [10, 12–20] that focused on the impact of DOX on the OS of glioma patients, with survival durations ranging from 6 to 18.5 months. Among these studies, 7 examined PFS, and 6 reported the median time-to-progression (mTTP). Response rates were also evaluated across all studies, ranging from 19 to 88%. In addition, the toxicity of DOX administration in glioma patients was a key aspect addressed in all of the reviewed literatures. The systematic review highlights the diverse impacts of DOX on glioma patients, primarily focusing on survival rates, with various studies showing promising outcomes in both OS and progression-free survival (Table 2).

**Table 2:** Characteristic table of included studies

First author	Year	Location	Study design	Sample type and size	WHO classification of glioma	mOS and PFS	mTTP	Gender	Mean age (years)	Response rate (%)	Doxorubicin dosage and administration details	Toxicity	Overall quality	Refs.
Beier et al.	2009	Germany	RCT	63 patients	Grade IV	mOS: 17.6 months PFS–12: 30.2%	NR	Male–female	NR	73	20 mg/m^2^ PEG–Dox + prolonged TMZ, plus radiotherapy	58 SAEs occurred, pulmonary embolism, and rapid decline in general condition	High	[10]
Pouillart et al.	1977	France	RCT	43 patients	Grade IV	mOS: 6 months	NR	Male–female	NR	72	Combination of Adriamycin, VM 26, and CCNU	Not specified	Low	[12]
Fabel et al.	2001	Germany	Case Series	15 patients	Grade IV	mOS: 10 months PFS-12: 15%	11 weeks	Male–female	NR	54	20 mg/m^2^ liposomal doxorubicin IV every 2 weeks	Low overall toxicity, skin toxicity, and myelosuppression	Moderate	[13]
Chua et al.	2004	Australia	Cohort	23 patients	Grade IV	mOS: 8.2 months PFS-6: 32% mPFS: 3.6 months	3.6 months	Male–female	55 years	19	200 mg/m^2^ TMZ orally (days 1–5), 40 mg/m^2^ Caelyx IV (day 1)	Well-tolerated, hematologic and non-hematologic toxicities reported	High	[14]
Hau et al.	2004	Germany	RCT	40 patients	Grade IV	mOS: 74 weeks PFS-6: 15% PFS-12: 7.5%	3.2 months	Male, female	NR	40	20 mg/m^2^ PEG–Dox with/without tamoxifen	Minor toxicity, skin toxicity, and myelosuppression	High	[15]
Glas et al.	2007	Germany	Case Series	63 patients	Grade III and IV	mOS: 8 months PFS-6: 27% PFS-12: 10%	NR	Male–female	NR	NR	PEG–DOX alone or with TMZ	WHO grade 4 myelotoxicity in 1 (2%), palmoplantar erythrodysesthesia	Moderate	[16]
Uzuka et al.	2006	Japan	Case Series	7 patients	Grade IV	mOS: 13.2 months	3.9 months	Male–female	NR	55.6	20 mg intra-arterial DOX during thermotherapy	Well-tolerated, facial flushing and alopecia	Moderate	[17]
Voulgaris et al.	2002	Greece	Case Series	10 patients	Grade III and IV	mOS: 39.9 weeks	39.83 weeks	Male–female	NR	60	0.5 mg intralesional DOX via Ommaya reservoir	No significant adverse reactions, bifrontal headache reported	Moderate	[18]
Ananda et al.	2011	Australia	Cohort	40 patients	Grade IV	mOS: 13.4 months PFS-6: 58%	6.2 months	Male–female	53 years	88	TMZ + PLD	Hematological toxicity: grade 3 neutropenia (8%), grade 3 non-hematologic toxicity (nausea, vomiting, and palmar–plantar toxicity)	High	[19]
Butt et al.	2021	USA	Case Series	30 patients	Grade IV	mOS: 13 months PFS-6: 27%	NR	Male–female	NR	NR	Combination of LITT with doxorubicin	Grade 1 (Headache, nausea, fatigue), grade 3 (hematologic leukopenia, neutropenia), grade 4 (neutropenia)	Moderate	[20]

*RCT* randomized clinical trial, *SAEs* serious adverse events, *mOS* median overall survival, *PFS-6* progression free survival at 6 months, *PFS-12* progression free survival at 12 months, *mPFS* median progression free survival, *NR* not reported, *mTTP* median time to progression

### Overall survival

The main focus of this systematic review is the duration of OS, ranging from 6 to 18.5 months. The first study selected for inclusion was conducted by P. Pouillart et al. in 1977, which involved the treatment of malignant gliomas in adults using a combination of Adriamycin, VM 26, and CCNU in a type II trial. This study, which included 43 patients, reported a median survival of 6 months with a maximum survival time of 17 months following the administration of DOX and the aforementioned drugs [12]. In our selected studies, Klaus Fabel and his colleagues were the first to use liposomal DOX to treat glioma patients in 2001. They treated 15 patients with 20 mg/m^2^ of liposomal DOX administered intravenously every 2 weeks. Their findings demonstrated that the MOS was prolonged, lasting 10 months after all and after the initiation of recurrence therapy, compared to OS in other phase II studies [13]. A recent study conducted in Greece involved 10 patients with malignant gliomas (type III and IV). Spyridoy Voulgaris et al. assessed the safety and effectiveness of administering DOX directly into the tumor. The patients received 0.5 mg of DOX in the Ommaya reservoir every 24 h for 10 days. Response evaluations showed that 6 out of 10 patients experienced clinical improvement lasting between 12 and 73 weeks. The median survival for all 10 patients was 39.9–45.52 weeks (range: 8–73 weeks). The study concluded that intralesional administration of DOX could be a safe and effective treatment for malignant gliomas [18]. In a phase 2 trial involving 23 patients with recurrent glioblastoma multiforme and a median age of 55, Susan L. Chua and colleagues administered a combination of temozolomide (200 mg/m2 orally, days 1–5) and Caelyx (40 mg/m2 i.v., day 1). The study found that MOS was 8.2 months (range, 1 to 16 + months) and concluded that the combination of temozolomide and Caelyx is well-tolerated, resulting in a modest objective response rate, and showing promising disease stabilization in the treatment of recurrent GBM [14]. Peter Hau and colleagues conducted a study in Germany to investigate the use of pegylated liposomal DOX (Caelyx^™^) in combination with and without tamoxifen on 40 patients with recurrent high-grade glioma. Each trial included twenty patients, and the study reported a MOS of 74 weeks from initial diagnosis and 26 weeks from the start of either Trial 1 or Trial 2 [15]. In a study conducted by Takeo Uzuka and colleagues in 2006, they included four male and three female Japanese individuals, with five cases of glioblastoma and two of anaplastic oligodendroglioma. The study utilized a new method involving the intra-arterial injection of Adriamycin DOX at a 20 mg dose via the common carotid artery during thermotherapy. The researchers reported that the drug was well-tolerated, and the median review, Martin Glas et al. conducted a Case Series to assess the effectiveness OS was 13.2 months [17]. In a retrospective of PEG–DOX as a standalone treatment or when combined with temozolomide. Their findings showed that the MOS after starting PEG–DOX (either as a single therapy or in combination) was 8 months (16 months for WHO III, and 7 months for WHO IV). They concluded that the use of PEG–DOX, with or without temozolomide, in treating recurrent malignant glioma was safe and moderately effective [16]. During a phase-I/II trial conducted in Germany, Christoph P Beier and colleagues examined the impact of PEG–Dox and extended administration of Temozolomide, in addition to radiotherapy, on 63 patients newly diagnosed with glioblastoma. In the phase-I portion of the study, PEG–Dox was administered using a 3-by-3 dose-escalation regimen, while in phase-II, patients received 20 mg/m^2^ of PEG–Dox once before radiotherapy and on days 1 and 15 of each 28-day cycle, starting 4 weeks after radiotherapy. The study yielded a MOS of 17.6 months, marking the highest reported OS among the literature we reviewed [10]. Sumitra Ananda and colleagues conducted a study to evaluate the effectiveness of using both TMZ and pegylated liposomal DOX (PLD) in treating individuals with newly diagnosed glioblastoma multiforme. Out of the 40 participants, with a median age of 53 and 73% being male, the study found that the OS was 13.4 months (95% CI 12.7–15.8 months) [19]. In our most recent reviewed research, Omar H. Butt and colleagues integrated DOX with laser interstitial thermal therapy (LITT) in thirty patients with recurrent glioblastoma. The study showed a substantial increase in OS to 13 months compared to historical controls who were treated with bevacizumab (*p* < 0.001) and LITT with standard salvage therapy (*p* < 0.05) [20]. The systematic review indicates that DOX, in various formulations and combinations, shows potential benefits for glioma treatment, with OS ranging from 6 to 18.5 months. Combination therapies, such as DOX with VM 26 and CCNU, yielded moderate success, while liposomal DOX significantly prolonged MOS to 10 months, particularly in recurrence therapy. Intralesional administration and novel combinations with temozolomide or laser interstitial thermal therapy (LITT) demonstrated improved survival rates, with Christoph P. Beier's trial reporting the highest MOS of 17.6 months. Despite promising outcomes, variability in response rates and the management of associated toxicities underscore the need for further research to optimize these treatment protocols.

### Progression-free survival and median time to progression

Out of the 7 studies we reviewed, all of them assessed the PFS in patients. PFS refers to"the duration of time in which a patient lives with a disease, such as cancer, without it progressing or worsening during and after treatment."This measure is used by researchers to gauge the impact of a drug or new treatment in managing the symptoms of an illness. Klaus Fabel et al. were the first to report a 15% PFS after 12 months [13]. Following this, Peter Hau's literature claimed that the 6-month and 12-month PFS rates were 15% and 7.5%, respectively [15]. Susan L. Chua and her colleagues in Australia reported that the median PFS for the group was 3.6 months, with a range of 1 to over 16 months. In addition, the 6-month PFS rate was 32% [14]. Glas and colleagues showed that PFS-6 and PFS-12 were 27% and 10%, respectively [16]. In their study, Christoph P Beier reported a PFS-12 of 30.2% [10]. The highest percentage of PFS was documented in Sumitra Ananda et al.'s study, with a PFS-6 of 58% [19]. Finally, in a 2021 study, Omar H. Butt demonstrated that the combined PFS at 6 months was 27% when both arms (Late + Early) were considered [20]. Within the realm of mTTP research, this approach is utilized to explore the duration from the initial diagnosis or commencement of treatment for an illness until the point at which the illness begins to deteriorate or spread to other areas of the body. When conducting a clinical trial, assessing TTP serves as a method to evaluate the effectiveness of a new treatment. In our research, the longest reported mTTP was 39.83 weeks in the study by Spyridoy Voulgaris et al. [18], while the shortest duration was 11 weeks as shown in Klaus Fabel's study [13]. In contrast, other researchers such as Peter Hau, Susan L. Chua, Takeo Uzuka, and Sumitra Ananda reported durations of 3.2 months, 3.4 months, 3.9 months, and 6.2 months, respectively [14, 15, 17, 19].

The reviewed studies consistently assessed PFS in glioma patients, highlighting the efficacy of various treatments in delaying disease progression. PFS, defined as the time during which a patient lives without disease worsening, serves as a crucial metric in evaluating treatment impact. Klaus Fabel et al. first reported a 15% PFS at 12 months, while Peter Hau documented 6-month and 12-month PFS rates of 15% and 7.5%, respectively. Susan L. Chua's study noted a median PFS of 3.6 months with a 6-month PFS rate of 32%. Glas et al. found PFS-6 and PFS-12 rates of 27% and 10%, respectively. Christoph P. Beier reported a PFS-12 of 30.2%, and Sumitra Ananda's study achieved the highest PFS-6 rate of 58%. Omar H. Butt's 2021 study demonstrated a combined 6-month PFS of 27%. mTTP also varied, with the longest being 39.83 weeks in Spyridoy Voulgaris et al.'s study, while the shortest was 11 weeks in Klaus Fabel's study. Other notable mTTP durations included 3.2 months, 3.4 months, 3.9 months, and 6.2 months from studies by Peter Hau, Susan L. Chua, Takeo Uzuka, and Sumitra Ananda, respectively. These findings underscore the variability and potential of different treatment protocols in extending PFS in glioma patients (Table 1).

### Response rate

The response rate is an essential measure indicating the proportion of survey respondents compared to the total sample size, with higher percentages reflecting more favorable responses. In this review, 9 out of 10 studies reported response rates, which varied significantly from a low of 19% to a high of 88%, with an average response rate of 56.8%. The lowest response rate of 19% was observed in the study by Susan L. Chua and colleagues, which involved administering a combination of temozolomide and Caelyx to patients with recurrent glioblastoma multiforme. In contrast, the highest response rate of 88% was reported in Sumitra Ananda's study, which evaluated the effectiveness of using both TMZ and pegylated liposomal DOX (PLD) in treating newly diagnosed glioblastoma multiforme patients. This significant variability in response rates across different studies highlights the diverse efficacy of treatment protocols. Factors contributing to this variability could include differences in patient demographics, tumor characteristics, and specific treatment regimens. These findings underscore the necessity for further research to refine and optimize dosing strategies and administration methods, aiming to enhance treatment outcomes and achieve more consistent response rates across patient populations. In addition, understanding the underlying reasons for the wide range of response rates could inform the development of personalized treatment approaches, ultimately improving the overall effectiveness of therapies for glioma patients (Table 1).

### Toxicity

The researchers in our selected studies observed a wide range of toxic effects following the administration of DOX, ranging from headaches to various hematological toxicities. However, almost all of them noted that this form of therapy was generally well-tolerated. Spyridoy Voulgaris stated that there were no significant adverse reactions either locally or systemically due to the intratumoral administration of DOX. Nevertheless, 4 out of 10 patients experienced a frontal headache during the drug injection. No other side effects, such as vomiting, fever, epileptic seizures, bleeding, or hair loss, were reported in their study [18]. In total, there were 58 serious adverse events (SAEs), and 9 of these SAEs were potentially linked to PEG–Dox as reported in the study by Christoph P Beier. Two patients passed away as a result of a potential treatment-related issue (one due to pulmonary embolism, and the other due to an unclear rapid decline in general condition); however, the overall safety profile remained positive [10]. Sumitra Ananda's research revealed that hematological toxicity, such as grade 3 neutropenia, was observed in 8% of cases. Non-hematologic toxicity, including nausea and vomiting (8%) and palmar–plantar toxicity (5%), was also observed. The study concluded that the combination of T and PLD was well-tolerated [19]. Hau work showed minor toxic effects, with the most frequent adverse reactions being skin irritation and decreased bone marrow function [15]. Omar H. Butt's article reported symptoms of grade 1 (including headache, nausea, and fatigue), grade 3 (such as hematologic leukopenia or neutropenia), and grade 4 (such as neutropenia) [20]. Klaus Fabel and his team reported that the overall toxicity of DOX in glioma patients was minimal. Throughout the treatment period until disease progression, the scores for quality of life and MMSE remained consistent. The most prevalent adverse effects observed in this study were skin toxicity and myelosuppression [13]. Takeo Uzuka reported that their protocol was well-received with only observed cases of facial flushing and alopecia [17]. On the other hand, Martin Glas and his team observed WHO grade 4 myelotoxicity in 1 patient (2%), grade 3 in 8 patients (16%), and grade 1–2 in 4 patients (8%). The most common nonhematologic toxicity observed was palmoplantar erythrodysesthesia [16]. The study by Susan L. Chua found that 18% of patients experienced grade 3/4 neutropenia and 18% experienced grade 3/4 thrombocytopenia. In addition, 14% of patients had grade 3 nonhematologic toxicity, including rash in 14% of patients, nausea and vomiting in 4% of patients, hypersensitivity reaction to Caelyx in 14% of patients, and palmar–plantar toxicity in 4% of patients. Despite these toxicities, the study concluded that the combination of temozolomide and Caelyx was well-tolerated [14].

The reviewed studies observed various toxic effects from DOX administration, including headaches and hematological toxicities, but generally found the therapy well-tolerated. Spyridoy Voulgaris reported minimal adverse reactions, with only headaches in 4 out of 10 patients. Christoph P. Beier noted 58 serious adverse events, including two deaths, but maintained a positive safety profile. Sumitra Ananda found grade 3 neutropenia in 8% of cases and non-hematologic toxicity in 13%, concluding the therapy was well-tolerated. Peter Hau reported minor skin irritation and decreased bone marrow function. Omar H. Butt recorded symptoms from grade 1 to grade 4, including neutropenia. Klaus Fabel found minimal overall toxicity, with only skin toxicity and myelosuppression. Takeo Uzuka noted facial flushing and alopecia. Martin Glas observed grade 4 myelotoxicity in 2% and palmoplantar erythrodysesthesia as common nonhematologic toxicity. Susan L. Chua found 18% experienced grade 3/4 neutropenia and thrombocytopenia but concluded the therapy was well-tolerated. Overall, DOX therapy was manageable and well-tolerated despite varied toxic effects (Table 1).

### Risk of bias in studies

RCT studies that we reviewed in this study were assessed with Cochrane Risk of Bias tool. The overall quality after assessment was low for Pouillart et al. [12] study and high for Hau et al. [15] and Beier et al. [10] studies [10, 12, 15]. The Case Series studies that included in our review were examined with JBI Checklist and the result of this assessment shown that all of these studies taken Moderate overall quality score [13, 16–18, 20]. We had two cohort articles in our literature and both of them taken high overall quality score in NOS Quality Assessment Tool [14, 19] (Table 3).

**Table 3:** Quality assessment of included studies

Study	Study design	Quality assessment tool	Selection bias	Performance bias	Detection bias	Attrition bias	Reporting bias	Other bias	Representativeness of cohort	Overall quality
Pouillart et al. [12]	RCT	Cochrane Risk of Bias	High	High	Unclear	Low	Low	High	Low	Low
Fabel et al. [13]	Case Series	JBI Checklist	Representative	N/A	Secure record	Yes	N/A	Independent blind	Yes	Moderate
Chua et al. [14]	Cohort	NOS	Somewhat representative	Drawn from same community	Secure record	Yes	Age, sex	Independent blind	Yes	High
Hau et al. [15]	RCT	Cochrane Risk of Bias	Low	Low	Low	Low	Low	Low	Low	High
Glas et al. [16]	Case Series	JBI Checklist	Representative	N/A	Secure record	Yes	N/A	Independent blind	Yes	Moderate
Beier et al. [10]	RCT	Cochrane Risk of Bias	Low	Low	Low	Low	Low	Low	Low	High
Uzuka et al. [17]	Case Series	JBI Checklist	Representative	N/A	Secure record	Yes	N/A	Independent blind	Yes	Moderate
Voulgaris et al. [18]	Case Series	JBI Checklist	Representative	N/A	Secure record	Yes	N/A	Independent blind	Yes	Moderate
Ananda et al. [19]	Cohort	NOS	Representative	Drawn from same community	Secure record	Yes	Age, sex	Independent blind	Yes	High
Butt et al. [20]	Case Series	JBI Checklist	Moderate	Low	Moderate	Moderate	Low	Moderate	Moderate	Moderate

*N/A* not assessed, *RCT* randomized clinical trial

## Discussion

Our systematic review sought to assess the impact of DOX on the life expectancy of individuals with glioma. After conducting a comprehensive search and selection process, we identified 10 studies that met our criteria. These studies differed in terms of their design, the characteristics of the patients involved, and the methods of administering DOX. The primary findings indicated a range of OS from 6 to 18.5 months. There was also significant variation in PFS and mTTP, underscoring the diverse nature of the included studies. While the toxicity profiles were generally manageable, some studies reported severe adverse effects. The OS rates observed in our review, which ranged from 6 to 18.5 months, suggest that while DOX has the potential to extend survival in glioma patients, its effectiveness varies widely depending on the method of administration and the characteristics of the patient population. For example, the study by Christoph P. Beier et al. reported the highest OS of 17.6 months using PEG–Dox in combination with radiotherapy and prolonged temozolomide administration [10]. This indicates that using combination therapies could potentially improve the efficacy of DOX. On the other hand, previous research, such as the study by P. Pouillart et al., which showed a 6-month OS, demonstrates the advancements in drug delivery and combination approaches over the years [12].

Our results align with prior research that emphasizes the difficulties and potential of utilizing DOX for treating gliomas. Research has consistently shown that while DOX is effective in treating various types of cancer, its effectiveness in brain tumors is hindered by the blood–brain barrier (BBB). Nevertheless, advancements in drug delivery systems, such as liposomal encapsulation and nano-carriers, have displayed potential in improving the delivery of DOX to the brain. Earlier studies have consistently highlighted the challenge of significantly enhancing OS in glioma patients using DOX. For example, early studies like Pouillart et al. [12] reported an OS of 6 months when utilizing a combination of DOX, VM 26, and CCNU, with a maximum of 17 months [12]. In 2001, Klaus Fabel and colleagues developed liposomal DOX, which resulted in a median OS of 10 months, representing a slight enhancement over conventional DOX formulations [13]. Additional research, including studies conducted by Voulgaris et al. [18] and Chua et al. [14], revealed different levels of success in terms of median OS values, with 39.9 weeks and 8.2 months, respectively. These findings indicate that the effectiveness of the treatment varies depending on the delivery method and its combination with other therapies [14, 18]. The longest OS recorded in our systematic review was 17.6 months in the study by Beier et al. [10], where they used PEG–Dox along with extended treatment of temozolomide and radiotherapy [10]. Emerging studies confirm these conclusions, highlighting the significant impact of DOX formulation and accompanying treatments on OS. For example, the use of DOX in combination with targeted shockwave therapy has demonstrated promise in increasing drug effectiveness and enhancing survival rates. However, the long-term advantages are still uncertain due to rapid tumor advancement after treatment [21]. The studies that were reviewed showed different results in terms of PFS. Initial findings, such as those from Fabel et al. [13] and Hau et al. [15], demonstrated PFS-6 rates of 15% and 7.5%, respectively, indicating the limited effectiveness of DOX in slowing down the progression of the disease [13, 15]. Newer research has shown increased PFS rates, for example, Ananda et al. [19] reported a PFS-6 of 58%, indicating considerable variation depending on the treatment protocols used [19]. Newer methods that use targeted delivery systems, like IL-13 receptor-targeted liposomal DOX, have demonstrated encouraging preclinical findings in animal models, indicating the possibility of enhanced clinical outcomes if these approaches can be successfully applied in human trials [22]. The response rates (RR) differed greatly among studies, with Chua et al. [14] reporting a low RR of 19% in their early work, while Ananda et al. [19] observed a high RR of 88% [14, 19]. DOX is typically well-tolerated in terms of toxicity, but common side effects include hematological toxicities and palmar–plantar erythrodysesthesia. Research conducted by Beier et al. [10] and Glas et al. [16] supported these results, suggesting that although the toxicity profiles can be managed, they are still an important factor to consider for clinical use [10, 16]. New advancements in drug delivery, such as focused shockwave therapy and targeted liposomal formulations, are designed to reduce the toxic effects of drugs by improving the accuracy of drug delivery to tumor sites. This in turn reduces the overall exposure to the drug and its associated side effects. The comprehensive review thoroughly examines a range of studies conducted over several decades, from 1973 to 2021. This long-term view provides a deeper understanding of the changing role of DOX in treating glioma. By encompassing a variety of clinical trials, observational studies, and retrospective analyses, the review presents a comprehensive assessment of DOX's effectiveness and safety across different research methodologies and patient groups. This diverse approach helps to identify consistent trends and outliers in the data. The thorough analysis thoroughly evaluates multiple important results, including OS, PFS, mTTP, response rate (RR), and side effect profiles. This comprehensive approach offers a detailed evaluation of how DOX affects patients with glioma. The review showcases research using new techniques, such as liposomal and PEGylated forms of DOX, administration directly into the tumor, and combining it with other chemotherapy drugs. These advancements are essential for overcoming traditional challenges such as the blood–brain barrier. By including studies from different parts of the world, especially a significant number from Germany, the review helps in understanding how treatment outcomes can vary globally. The scope of the study is limited due to the inclusion of studies with diverse designs, patient groups, and treatment plans, resulting in significant variation. This diversity makes it difficult to directly compare findings and reach definitive conclusions about the effectiveness of DOX. Many of the studies have small sample sizes, with some involving as few as 7 to 63 patients. These limited sample sizes reduce the statistical strength of the results and the ability to apply the findings to the wider glioma patient population. Variations in dosing schedules, treatment combinations, and methods of administration (such as intravenous, intra-arterial, and intralesional) across studies complicate the interpretation of the data. Standardization in these areas is essential for more dependable comparisons and conclusions. The systematic review may be influenced by publication bias, where studies with positive results are more likely to be published than those with negative or neutral findings. This bias could affect the overall evaluation of d DOX's effectiveness. Some of the studies included in the review have limited follow-up periods, which makes it challenging to assess the long-term effectiveness and safety of DOX in glioma patients. Long-term data is crucial for understanding the lasting impact of treatment and potential delayed side effects. Variations in how outcomes such as OS, progression-free survival, and toxicity are defined and reported across studies make direct comparisons complex. Using uniform criteria and standardized reporting protocols would improve the consistency and reliability of the results. The variation seen in our analysis can be explained by a number of factors. Variances in patient characteristics, glioma severity, and treatment approaches all play a role in the differences in results. For instance, studies that employed advanced drug delivery systems such as PEG–Dox tended to show improved outcomes compared to those using conventional administration techniques. Subgroup analyses, such as comparing outcomes between different grades of glioma or administration approaches, were constrained by the limited number of studies and inconsistent reporting criteria. Based on our results, it is indicated that DOX, especially when utilized in advanced delivery methods or combined treatments, may have the potential to enhance survival rates in patients with glioma. Healthcare providers should contemplate the utilization of liposomal DOX or combined treatment plans to improve drug delivery and effectiveness. The findings of this systematic review highlight the potential benefits and challenges associated with DOX use in glioma treatment. The extended OS observed in studies utilizing liposomal and PEGylated DOX formulations suggests that advanced drug delivery methods may improve treatment efficacy by enhancing blood–brain barrier penetration. Combination therapies, such as DOX with temozolomide or radiotherapy, also demonstrated promising survival outcomes. Given these mixed outcomes, further well**-**structured, large-scale clinical trials are essential to determine the optimal DOX formulation, dosing schedule**,** and combination therapy strategies. Standardizing outcome measures and reporting methods will also enhance comparability across future studies.

Nevertheless, due to the inconsistency in results and the possibility of severe side effects, it is important to carefully select and monitor patients. Future studies should prioritize the following areas: (1) Conducting larger trials across multiple centers with standardized protocols to validate the results and minimize variation. (2) Investigating and improving advanced methods of drug delivery to overcome the blood–brain barrier and improve drug effectiveness. (3) Researching the long-term effects and influence on quality of life to offer a thorough evaluation of the usefulness of DOX in treating glioma.

## Conclusion

Our systematic review emphasizes the potential of DOX in enhancing survival rates for glioma patients, especially when utilized with advanced delivery techniques or in combination with other therapies. This systematic review underscores the potential role of DOX in glioma treatment, particularly when used in liposomal or PEGylated forms and in combination with other therapies. Despite promising survival benefits, the variability in study results and concerns regarding toxicity highlight the need for further high-quality clinical research. Future investigations should focus on overcoming the blood–brain barrier limitations, optimizing drug delivery methods, and evaluating long-term patient outcomes to establish DOX as a viable treatment option for glioma patients.

## Data Availability

No datasets were generated or analysed during the current study.
